# PROTOCOL: The Application of Information and Communication Technologies in Physical Activity Interventions for Breast Cancer: A Scoping Review Protocol

**DOI:** 10.1002/cl2.70044

**Published:** 2025-06-02

**Authors:** Xin Chen, Maaz Imam, Yutong Yi, JJ Pionke, Lixcy Vega, Anna Arthur, Jessie Chin, Chungyi Chiu

**Affiliations:** ^1^ Department of Dietetics and Nutrition University of Kansas Medical Center Kansas City Kansas USA; ^2^ Carle Illinois College of Medicine Urbana Illinois USA; ^3^ Department of Food Science and Human Nutrition University of Illinois Urbana‐Champaign Urbana Illinois USA; ^4^ iSchool Syracuse University Syracuse New York USA; ^5^ School of Information Sciences University of Illinois Urbana‐Champaign Champaign Illinois USA; ^6^ Department of Health and Kinesiology University of Illinois Urbana‐Champaign Urbana Illinois USA

## Abstract

This is the protocol of a Campbell scoping review. This scoping review aims to identify and map the evidence regarding the use of information and communication technologies (ICTs) in physical activity (PA) interventions for breast cancer survivors (BCS), which includes examining the types of ICTs utilized, how they are applied, and their effects on BCS' PA‐related outcomes. The Preferred Reporting Items for Systematic Reviews and Meta‐Analysis extension for Scoping Reviews Checklist (PRISMA‐ScR) was used to structure this protocol. The focus of the scoping review is guided by the mnemonic PCC (Population, Concept, Context) recommended by the JBI Manual for Evidence Synthesis. PubMed, CINAHL (Ebsco), Web of Science (Clarivate), and SportDiscus (Ebsco) will be searched for peer‐reviewed studies. We will include interventional studies using ICTs for PA promotion among BCS across the cancer care continuum. Our protocol incorporates information about the aims and importance of the scoping review, search strategy, inclusion/exclusion criteria, study selection, data extraction, quality assessment, and data synthesis. The total number and key characteristics of included studies will be reported in this scoping review. It will identify and map the current ICTs used in PA interventions, outline the methods and extent of their application, and summarize the related outcomes observed among BCS. Participation in PA remains insufficient among BCS despite its known benefits in lowering the risk of death and improving breast cancer prognosis. While technology‐based interventions have received increased attention in recent years, there is still limited consensus within the scientific literature surrounding ICT‐based PA interventions for BCS. The use of ICTs in PA interventions may promote PA among BCS and benefit their survivorship. This scoping review may lead to strategies for developing ICTs that are optimal to be used in PA interventions that benefit BCS, a large and growing population of cancer survivors. Additionally, it will identify knowledge gaps to enhance healthcare communication between healthcare practitioners and BCS using ICTs, which may promote PA and lead to improved cancer survivorship in this population. Registered and available at OSF (https://osf.io/hwde4/?view_only=be6ef619dc1a44b080bb61be32d5d56f).

## 1 Background

### 1.1 Introduction

Cancer is a significant public health problem worldwide, with breast cancer being the leading cause of cancer mortality for women globally as of 2022 (Wilkinson and Gathani 2022). The National Cancer Institute defines a cancer survivor as anyone with a history of cancer, from the time of diagnosis through the remainder of life (National Cancer Institute n.d.). For women, breast cancer alone accounts for almost one‐third of female cancers (Siegel et al. 2023). It is a growing concern worldwide, with an increasing incidence rate over the past few decades (Wilkinson and Gathani 2022). In addition, the population of breast cancer survivors (BCS) has continued to grow. This trend can be attributed to a combination of factors, such as the growing aging population, advancements in early detection, and more effective treatments, such as targeted therapies, resulting in improved cancer survival rates (Miller et al. 2022). Evidence shows that BCS caregivers who perform various caregiving tasks experience considerable physical and emotional stress, which can persist for several years beyond BC treatments (Wagner et al. 2011). The above findings highlight the need for healthcare professionals (e.g., nurses) to acknowledge the unique challenges faced by BCS and their caregivers and provide them with relevant support. Physical activity (PA) benefits not only the general population but also provides various advantages for BCS, including reducing the risk of breast cancer‐related death and recurrence, preventing muscle loss, improving physical function, enhancing the quality of life, weight management, and disease‐related fatigue (Agussalim et al. 2024; Zeng et al. 2014; Berger et al. 2015; Bower et al. 2014). Furthermore, behavioral change monitoring strategies used in PA have been shown to improve health‐related outcomes among the population (Duijts et al. 2011). However, a physically active lifestyle meeting the public health standard still needs to be promoted among cancer survivors, as approximately 66.5% of cancer survivors do not meet or follow the PA guidelines (Troeschel et al. 2018). Specifically, while 65%–75% of BCS were classified as capable of participating in a PA program according to clinical guidelines (Igwebuike et al. 2018), only 20%–45% adhered to the recommended guidelines (Campbell et al. 2019; Coletta et al. 2019; Mason et al. 2013; Smith et al. 2018). The role of healthcare providers has expanded to include health coaching in PA and the management of chronic diseases (Douglas et al. 2006). However, despite the importance of PA for BCS, many BCS remain insufficiently physically active after cancer diagnosis (Avancini et al. 2020). Thus, there is a strong need for us to understand how to facilitate long‐term survivorship care for BCS that incorporates PA to help manage disease symptoms and improve their survivorship or quality of life.

There has been a rapid increase in the availability of advanced healthcare technologies, which have extended and improved the quality of life of people (Wamble et al. 2019), including BCS. Since 2003, online communication has significantly increased between healthcare recipients and providers (Tarver et al. 2018). Communication technology applied in healthcare enables outreach among people effectively despite physical distance, offering benefits for both healthcare practitioners and their patients (Ryan et al. 2015). Furthermore, technology may improve access to information and services, promote behavior change, and enable remote monitoring and support (Free et al. 2013), with evidence supporting its role in managing chronic diseases (e.g., breast cancer) (Fan and Zhao 2022).

Information and Communication Technology (ICT) is a broad term with various applications. For example, digital health is one type of ICT application in healthcare (Ronquillo et al. 2023). Within the context of this review, ICT is defined as any form of technology (e.g., digital, wired, or wireless electronic devices, computer programs, mobile apps) capable of transmitting information or facilitating communication regarding self‐care management between healthcare recipients (e.g., cancer survivors) and healthcare professionals (Chetley et al. 2010; World Health Organization n.d.). The COVID‐19 pandemic accelerated the use of ICT in healthcare (Wong et al. 2021). The temporary shutdown of healthcare facilities and fear of exposure to COVID‐19 delayed cancer diagnosis and treatment, driving a shift to digital solutions and adjusting people to a “new normal” that involves increased usage of ICTs (Patt et al. 2020; Lee and Lee 2021). This context illustrates the growing interest in and reliance on virtual approaches in healthcare for people in recent years. ICT has the potential to support the implementation and spread of practices that promote positive behavior change, including PA. For example, accessing PA programs through ICTs (e.g., mobile apps, email, and phone calls) can encourage BCS's participation in PA and improve compliance (Martin et al. 2021). As the population of BCS continues to grow, this population may benefit from ICTs that provide PA promotion and guidance (Wamble et al. 2019). In sum, PA improves outcomes in BCS, while ICTs support PA by enhancing delivery, guidance, and motivation, leading to better adherence to behavior change that benefits overall health. This underscores the importance of healthcare providers adopting ICT‐based PA interventions to improve survivorship in BCS. Thus, it is valuable to review the types, how, and to what extent ICTs have been applied to promote PA among BCS.

The current review is important because it will advance our understanding of the implementation and dissemination of evidence‐based PA interventions delivered with the help of ICT targeting BCS (Nekhlyudov et al. 2020; NCI DCCPS 2020). ICT‐based interventions have been shown to be effective and helpful in easing the burden of caregivers (Lindberg et al. 2013), which aligns with addressing the need for continued investment and promotion in healthcare innovation to further improve oncological care for BCS. The current Survivorship Care Plans are recommended tools for cancer care professionals to guide cancer care, but often focus on the acute treatment phases, resulting in a gap in long‐term cancer survivorship (Elizondo Rodriguez et al. 2022). To promote continued care across all stages of cancer survivorship, it has been recommended to enhance communication among different stakeholders in cancer care and build a comprehensive cancer survivorship program that includes patients of different stages (Douglas et al. 2006; Flores et al. 2019). To make sure we have a comprehensive inclusion of BCS in this review protocol, we acknowledge two critical subgroups: (1) BCS undergoing active treatment (e.g., chemotherapy, radiotherapy) and/or with acute disease (BCS‐AT), as well as (2) BCS who have completed certain cancer treatments in the past and are in long‐term survivorship or care (BCS‐LS). A review with such a subgroup analysis approach could be helpful in (1) understanding if each subgroup has unique needs based on their stage of treatment or survivorship and (2) identifying the existing ICT‐based PA intervention across the breast cancer continuum.

### 1.2 Why a Scoping Review?

The concept of ICT is a domain that is relatively new, rapidly evolving, and lacking in consensus on best practices. The application of ICTs in this context is broad and heterogeneous, with various formats and technologies being utilized. This diversity makes it challenging to determine optimal approaches or draw consistent conclusions about the usage and impact of ICTs on PA outcomes among BCS. Although a variety of distinct studies surrounding PA, breast cancer, and healthcare delivery technology and applications exist (Delrieu et al. 2020; Galiano‐Castillo et al. 2013; Yang et al. 2011; Dorri et al. 2020), the scientific literature presents mixed results regarding the types of ICTs used in PA interventions and their effects. Review studies explicitly focusing on technology defined as ICTs remain limited. Moreover, contextual factors such as cancer stage, treatment phase, and demographic differences were not always considered when evaluating the outcomes of ICT‐based PA interventions. All these knowledge gaps underscore the need for a scoping review to systematically map the existing evidence, identify effective ICT strategies for PA promotion among BCS, and explore areas where further research is needed.

Scoping reviews are designed to identify and map diverse evidence, tools, characteristics related to a concept, and knowledge gaps; it is suitable when the literature spans different concepts, research methodologies, and intervention formats (Peters et al. 2021). While systematic reviews focus on assessing the effectiveness of specific interventions, scoping reviews allow for a comprehensive overview beyond effectiveness and answer complex questions where comparing interventions may be neither relevant nor possible (Munn et al. 2018). Thus, based on the broad scope and emerging nature of this field, where best practices still need to be established, a scoping review is likely the most appropriate review type for the current review purpose. The results of the scoping review will assist in deciding if a systematic review is further needed, for example, examining the effectiveness of an ICT‐mediated PA intervention for a specific stage of cancer care (Khalil and Tricco 2022).

## 2 Objectives

The aim of this review is to identify and map the evidence on the use of ICTs in PA interventions for BCS, including but not limited to what ICTs are used, how they are applied, and their impact on PA promotion among the population. The main objectives are the following:

1. To identify and map existing ICTs applied in PA interventions for BCS.
2. To summarize practice strategies based on reported changes in PA‐related outcomes of the ICT‐based interventions.
3. To identify and evaluate the current research gap and inform future research directions.

## 3 Methods

### 3.1 Design

We worked with a librarian (the fourth author) with expertise in conducting systematic reviews to develop and conduct searches for this scoping review. We will use the mnemonic PCC (Population, Concept, Context) framework to guide the scope and set up our inclusion and exclusion criteria (JBI 2024; Khalil and Tricco 2022). We will follow the Preferred Reporting Items for Systematic Reviews and Meta‐Analysis Extension for Scoping Reviews (PRISMA‐ScR) Checklist, which is a PRISMA extension for scoping reviews, to prepare and conduct the current review (Tricco et al. 2018). This scoping review will comprise a literature search, article selection, data extraction, quality appraisal, data analysis, and data synthesis.

### 3.2 Search Strategy and Sources

We conducted a literature search in the following databases: PubMed, CINAHL (Ebsco), Web of Science (Clarivate), and SportDiscus (Ebsco). We will also hand search for other evidence, including gray literature, dissertations, trial registries, reference lists from other reviews, articles found in prominent oncology journals, or using web search engines such as Google Scholar to ensure we identify as much relevant evidence as possible. Key terms related to our study's interest are extracted from an initial review of the literature. We will develop, improve, and adapt the search terms iteratively to finalize our search within the above databases. Search terms will include appropriate Medical Subject Headings (MeSH) terms and keywords related to ICTs, breast cancer, PA, and terms specific to the treatment status for the separate analysis based on the two mentioned subgroups. The database‐categorized search terms we used in the review for the subgroup BCS‐AT (i.e., BCS with active treatment) are shown in Table 1, 3. The search terms that will be used for the subgroup BCS‐LS (i.e., BCS who have completed primary treatment in the past and are in long‐term survivorship or care) are included in Table 2 and categorized by the database.

**Table 1:** Search terms for subgroup BCS‐AT (breast cancer survivors with active treatment).

*PubMed:*
1. (“breast cancer” OR “Breast Neoplasms”[Mesh] OR “Breast Neoplasms”) 2. #1 AND (“current treatment” OR “under treatment” OR “Patients”[Mesh] OR “Patients” OR “Outpatients”[Mesh] OR “Outpatients”) 3. #1 AND #2 AND (“physical activity”[Mesh] OR “physical activity” OR “Exercise”[Mesh] OR “Exercise” OR “physical fitness” OR “physical fitness”[Mesh]) 4. #1 AND #2 AND #3 AND (“ICT” OR “Telemedicine”[Mesh] OR “Telemedicine” OR “telehealth” OR “telehealth”[Mesh] OR “Mobile Health”[Mesh] OR “Mobile Health” OR “Information Technology”[Mesh] OR “Information Technology” OR “Software”[Mesh] OR “Software” OR “Mobile Applications”[Mesh] OR “Mobile Applications” OR “Internet‐Based Intervention”[Mesh] OR “Internet‐Based Intervention” OR “world wide web applications” OR “world wide web applications”[Mesh] OR “Social Media”[Mesh] OR “Social Media” OR “Internet”[Mesh] OR “Internet”)
*CINAHL:*
1. (“breast cancer” OR “Breast Neoplasms”[Mesh] OR “Breast Neoplasms”) 2. #1 AND (“current treatment” OR “under treatment” OR “Patients”[Mesh] OR “Patients” OR “Outpatients”[Mesh] OR “Outpatients”) 3. #1 AND #2 AND (“physical activity”[Mesh] OR “physical activity” OR “Exercise”[Mesh] OR “Exercise” OR “physical fitness” OR “physical fitness”[Mesh]) 4. #1 AND #2 AND #3 AND (“ICT” OR “Telemedicine”[Mesh] OR “Telemedicine” OR “telehealth” OR “telehealth”[Mesh] OR “Mobile Health” OR “Information Technology”[Mesh] OR “Information Technology” OR “Software”[Mesh] OR “Software” OR “Mobile Applications”[Mesh] OR “Mobile Applications” OR “Internet‐Based Intervention”[Mesh] OR “Internet‐Based Intervention” OR “world wide web applications” OR “world wide web applications”[Mesh] OR “Social Media”[Mesh] OR “Social Media” OR “Internet”[Mesh] OR “Internet”)
*Web of Science:*
1. TS=(“breast cancer” OR “Breast Neoplasms”) 2. #1 AND TS=(“current treatment” OR “under treatment” OR “Patients” OR “Outpatients”) 3. #1 AND #2 AND TS=(“physical activity” OR “Exercise” OR “physical fitness”) 4. #1 AND #2 AND #3 AND TS=(“ICT” OR “Telemedicine” OR “telehealth” OR “Mobile Health” OR “Information Technology” OR “Software” OR “Mobile Applications” OR “Internet‐Based Intervention” OR “world wide web applications” OR “Social Media” OR “Internet”)
*SportDiscus:*
1. (“breast cancer” OR “Breast Neoplasms”) 2. #1 AND (“current treatment” OR “under treatment” OR “Patients” OR “Outpatients”) 3. #1 AND #2 AND (“physical activity” OR “Exercise” OR “physical fitness”) 4. #1 AND #2 AND #3 AND (“ICT” OR “Telemedicine” OR “telehealth” OR “Mobile Health” OR “Information Technology” OR “Software” OR “Mobile Applications” OR “Internet‐Based Intervention” OR “world wide web applications” OR “Social Media” OR “Internet”)

**Table 2:** Search terms for subgroup BCS‐LS (i.e., breast cancer survivors who have completed primary treatment in the past and are in long‐term survivorship or care).

*PubMed:*
1. (“breast cancer” OR “Breast Neoplasms”[Mesh] OR “Breast Neoplasms”) 2. #1 AND (“Survivors”[Mesh] OR “Survivors” OR “Cancer Survivors”[Mesh] OR “Cancer Survivors”) 3. #1 AND #2 AND (“physical activity”[Mesh] OR “physical activity” OR “Exercise”[Mesh] OR “Exercise” OR “physical fitness” OR “physical fitness”[Mesh]) 4. #1 AND #2 AND #3 AND (“ICT” OR “Telemedicine”[Mesh] OR “Telemedicine” OR “telehealth” OR “telehealth”[Mesh] OR “Mobile Health”[Mesh] OR “Mobile Health” OR “Information Technology”[Mesh] OR “Information Technology” OR “Software”[Mesh] OR “Software” OR “Mobile Applications”[Mesh] OR “Mobile Applications” OR “Internet‐Based Intervention”[Mesh] OR “Internet‐Based Intervention” OR “world wide web applications” OR “world wide web applications”[Mesh] OR “Social Media”[Mesh] OR “Social Media” OR “Internet”[Mesh] OR “Internet”)
*CINAHL:*
1. (“breast cancer” OR “Breast Neoplasms”[Mesh] OR “Breast Neoplasms”) 2. #1 AND (“Survivors”[Mesh] OR “Survivors” OR “Cancer Survivors”[Mesh] OR “Cancer Survivors”) 3. #1 AND #2 AND (“physical activity”[Mesh] OR “physical activity” OR “Exercise”[Mesh] OR “Exercise” OR “physical fitness” OR “physical fitness”[Mesh]) 4. #1 AND #2 AND #3 AND (“ICT” OR “Telemedicine”[Mesh] OR “Telemedicine” OR “telehealth” OR “telehealth”[Mesh] OR “Mobile Health”[Mesh] OR “Mobile Health” OR “Information Technology”[Mesh] OR “Information Technology” OR “Software”[Mesh] OR “Software” OR “Mobile Applications”[Mesh] OR “Mobile Applications” OR “Internet‐Based Intervention”[Mesh] OR “Internet‐Based Intervention” OR “world wide web applications” OR “world wide web applications”[Mesh] OR “Social Media”[Mesh] OR “Social Media” OR “Internet”[Mesh] OR “Internet”)
*Web of Science:*
1. TS=(“breast cancer” OR “Breast Neoplasms”) 2. #1 AND TS=(“Survivors” OR “Cancer Survivors”) 3. #1 AND #2 AND TS=(“physical activity” OR “Exercise” OR “physical fitness”) 4. #1 AND #2 AND #3 AND TS=(“ICT” OR “Telemedicine” OR “telehealth” OR “Mobile Health” OR “Information Technology” OR “Software” OR “Mobile Applications” OR “Internet‐Based Intervention” OR “world wide web applications” OR “Social Media” OR “Internet”)
*SportDiscus:*
1. (“breast cancer” OR “Breast Neoplasms”) 2. #1 AND (“Survivors” OR “Cancer Survivors”) 3. #1 AND #2 AND (“physical activity” OR “Exercise” OR “physical fitness”) 4. #1 AND #2 AND #3 AND (“ICT” OR “Telemedicine” OR “telehealth” OR “Mobile Health” OR “Information Technology” OR “Software” OR “Mobile Applications” OR “Internet‐Based Intervention” OR “world wide web applications” OR “Social Media” OR “Internet”)

We will record the search process (including the sources searched, when, by whom, and using what terms) in Word documents. All the articles identified from the searches of the relevant databases will be stored within the citation‐organizing software Zotero. This will further enable the research team to remove any duplicates.

### 3.3 Inclusion Criteria

The following PCC framework is based on the study aim and will help clarify the review's scope while guiding the inclusion criteria.

- Population: The population will include people aged 18 years or above who have been diagnosed with ductal carcinoma in situ or Stages I–IV breast cancer. The population must have undergone/currently be undergoing a PA intervention mediated by or based on any type of ICTs.
- Concept: The concept focuses on ICTs that can transmit information and facilitate communication between participants and researchers/health professionals in any type of PA interventions for health outcome improvement, including but not limited to (1) exercise counseling, (2) individual or group exercise programs, and (3) monitored home‐based exercise sessions. Outcomes of interest include the types of ICTs used, their usability, feasibility, adherence, and impact on PA‐related outcomes (e.g., behavior change, self‐reported or measured health outcomes, motivation or self‐efficacy in PA, and other key performance indicators reported).
- Context: The context considers studies from diverse settings utilizing ICTs in PA interventions for BCS. Key contextual factors include geographic elements, such as the recruitment country. The review will also examine racial, ethnic, and gender‐based considerations that affect PA intervention participation. Additionally, it will account for other characteristics of BCS, including treatment status (pretreatment, active, posttreatment), breast cancer stage (early vs. advanced), and intervention setting (home‐based vs. clinical).

For the literature search, all study types without restrictions on the year of publication will be included, and search terms tailored to each subgroup will be used to capture a broad scope of the literature. Only papers published in English will be reviewed. For the final full‐text review stage, we will focus on relevant peer‐reviewed interventional studies (e.g., randomized controlled trials, nonrandomized controlled clinical trials, pre‐post studies) using ICTs. Reviews, editorials, and commentaries will be included for review only if they explicitly address the relevant topic.

### 3.4 Exclusion Criteria

We will exclude studies that were not published in English and not empirical in nature, as well as case studies without empirical data. Studies will also be excluded if they focus on PA interventions without any ICT applied. We will exclude non‐English articles due to resource limitations in translating and verifying the accuracy and the predominance of relevant literature available in English, ensuring consistency and minimizing potential biases from translation errors.

### 3.5 Study Selection

One reviewer will organize all the articles identified after removing duplicates and arrange to assign weekly screening tasks among reviewers. Three reviewers will independently screen all article titles and abstracts weekly to check the identified articles' potential eligibility against the inclusion criteria, exclusion criteria, and relevancy, and take notes about the justifications of their decision, resulting in three sets of screening results. Full texts of primary studies may be reviewed in situations where it is uncertain whether a specific article meets eligibility. One reviewer will collect the screening results separately from each reviewer and compile them into an Excel spreadsheet each week for discussion. Then, the reviewers will discuss any discrepancies until an agreement is reached to include or exclude the studies. Reasons for the inclusion and exclusion of the articles will be noted. All screening results will be coded and input into an Excel spreadsheet for inter‐coder reliability checking. Research team members will also conduct relevant inter‐coder reliability calculations through the Reliability Calculator for three or more Coders (ReCal3) (Freelon 2023), which tracks the extent to which the independent coders agree on the selection of the abstracts based on the inclusion and exclusion criteria. We will aim to achieve inter‐coder reliability at 0.80. We will include the studies that meet the inclusion criteria and exclude those that fail to meet the inclusion criteria for data extraction. Two independent reviewers will then review the full text of the selected studies to ensure they meet the inclusion criteria. If agreement cannot be reached, a third reviewer (the first author) will continue to examine the reviewed studies' full text to make the final decision on whether to include the study for full‐text review or not. If disagreement cannot be solved in study selection, a group discussion consulting with the study supervisor (the last author) will be provided. A PRISMA‐ScR flow diagram of the literature search and selection process will be employed to record the details of the process and ensure that the study can be reproduced.

### 3.6 Data Extraction and Charting

Extractors will be trained to independently extract the data from the full text of the selected articles using a standardized Excel spreadsheet that includes all relevant domains for extraction. The extractors will not be informed about or discuss the details of our study's objectives or potential findings to reduce the risk of bias that may be introduced by their prior knowledge, experience, or expectations influencing the data extraction process. To ensure extraction reliability, data extraction will be conducted independently in duplicate for each article. A primary reviewer will perform the initial extraction, followed by a second reviewer who will independently extract the same data and then compare the results to identify discrepancies. Any discrepancies or errors that need to be resolved will be discussed within the research team to ensure that the extracted data is complete, accurate, and consistent. A summary table (Table 1, 3) will be presented to show the information and extracted data of the articles in the following domains: (1) basic information of the articles (year, author, title, journal), (2) study design (country of study, purpose; methods; sample size; statistical method), (3) subject characteristics (including age, race, ethnicity, breast cancer stage at diagnosis, and phase of the cancer continuum such as treatment status), and (4) intervention: [4.a.] ICT (e.g., ICT type, characteristics, usage frequency, and duration); [4.b.] PA program (e.g., intervention type and structure; protocols of the control group and experimental group), and (5) evaluation (e.g., outcome measures, statistical significance of outcome results, and other key performance indicators reported). If a study is included with more than two intervention arms, we will include in the review only the information about the intervention and control groups that meet the eligibility criteria. Missing data will be considered within the quality assessment, but authors will not be contacted due to time constraints.

**Table 3:** Summary Table Example.

No.	Author	Year	Country	Title	Study purpose	Study design	Subject demographics	ICT	Physical activity	Group comparison	Outcome measurements	Results	Extra notes
	(Last name of the first author, et al.)	2024	USA				Sample Size: Mean Age: Gender: Breast Cancer: Race: Ethnicity: Treatment status:	Type: Frequency: Follow‐up: Duration:	Type: Intensity: Frequency: Duration:	*Intervention*:(Sample size (*N*) + Protocol) *Control*: (Sample size (*N*) + Protocol)	(e.g., The Godin‐Shephard leisure‐time physical activity questionnaire)	*Significant*: (1) Y* (e.g., *p* < 0.05); (2) Y** (e.g., *p* < 0.01) *NS* (nonsignificant change) *Somewhat*: *SW* (significant change “within” group across time points)	Any other notes from the articles that are worth noting

### 3.7 Data Synthesis and Analysis

Two members of the research team will take part in the data synthesis and analysis. The extracted data will be collated by the third member and then summarized using descriptive analysis that maps out the results of the reported outcomes, key themes, and the relationships in the data. We will discuss and compare the current research findings available to help provide a comprehensive overview of the scope. The study objectives and methodologies of the included articles will be assessed to determine whether the study results are comparable across studies. This step helps ensure that the illustration of data analysis is appropriate and informs future research directions. With that, standardized codes (e.g., “Not at all,” “Somewhat,” or “Significant”) will be developed to help categorize and document the reported effects, impact, or changes based on the PA measurements resulting from an ICT‐based intervention. The PA measurement examples will include but are not limited to, improvements in PA outcomes, frequency, duration, physical functioning, and motivation. “Significant” will be coded only when a reported effect or change in the ICT‐intervention group is significant compared to the comparator or control group. “Somewhat” will be coded if there is a significant difference over time within the intervention group but not between groups, or if a significant change is observed only in a subgroup of the population. “Not at all” will be coded if the results show that an ICT‐based PA intervention barely changes any PA‐related outcomes. After synthesizing the reviewed studies, we will discuss the current state of ICTs, including their feasibility, usability, impact, and limitations in their application in PA interventions for BCS in cancer care. This aligns with the exploratory nature of scoping reviews, which aim to map evidence, summarize findings, and inspire future research directions (e.g., as a precursor to determining the need for a systematic review).

### 3.8 Evidence Grouping

The breadth of the scope of this review implies a likelihood of high diversity in the data reported by various studies. To understand the diversity and categorizing studies based on key themes, we will explore and identify potential sources through the following pre‐determined subgroups: (1) reported outcomes, (2) types of ICTs, (3) types of PA interventions, (4) breast cancer treatment statuses (pre‐, under‐, post‐, or in long‐term care), (5) cancer stages, (6) intervention follow‐up time points. If the final selected studies allow, separate analyses using suitable statistical tests (e.g., mean difference analysis) may be conducted based on the above subgroups to explore further and describe the evidence of ICTs in PA interventions among BCS.

## Author Contributions

Xin Chen and Chungyi Chiu conceived the topics and designed the protocol. Xin Chen, JJ Pionke, and Chungyi Chiu collaborated to develop and improve the search strategies and sources. Xin Chen, Maaz Imam, and Chungyi Chiu are involved in the protocol drafting process. All the authors are involved with protocol review and revisions. Chungyi Chiu, along with Xin Chen, gave the final check and approval for the protocol.

## Conflicts of Interest

The authors declare no conflicts of interest.

## Sources of Support

*Internal sources*

Funding Source 1, USA

University of Illinois Urbana‐Champaign. The Social and Behavioral Sciences Research Initiative (SBSRI) Small Grants Program (ID#200250): “Exploring Patient‐Centered e‐Intervention of Dual Health Behaviors among Breast Cancer Patients and Survivors.”

Funding Source 2, USA

University of Illinois Urbana‐Champaign Center for Social and Behavioral Sciences Seed Grant and USDA‐NIFA Hatch Project 1011487.

*External sources*

No sources of support were provided.
